# Supplementary material for: The SUMOylation Pathway Components Are Required for Vegetative Growth, Asexual Development, Cytotoxic Responses, and Programmed Cell Death Events in Fusarium oxysporum f. sp. niveum
Source: J Fungi (Basel). 2023 Jan 9;9(1):94. doi: 10.3390/jof9010094 (PMC9866417; doi:10.3390/jof9010094)
Supplement: Supplementary file 1 [file jof-09-00094-s001.zip › jof-2140474 - Supplementary Materials.pdf]

Supplementary Material

The SUMOylation Pathway Components are Required for Vegetative Growth, Asexual Development, Cytotoxic Responses, and Programmed Cell Death Events in *Fusarium oxysporum* f. sp. *niveum*

Azizullah [1,2], Muhammad Noman [1,2], Yizhou Gao [1,2], Hui Wang [1,2], Xiaohui Xiong [1,2], Jiajing Wang [1,2], Dayong Li [1,2,*] and Fengming Song [1,2,*]

[1] Ministry of Agriculture Key Laboratory of Molecular Biology of Crop Pathogens and Insect Pests, Institute of Biotechnology, Zhejiang University, Hangzhou, Zhejiang 310058, China

[2] Key Laboratory of Biology of Crop Pathogens and Insects of Zhejiang Province, Institute of Biotechnology, Zhejiang University, Hangzhou, Zhejiang 310058, China

* Correspondence: dyli@zju.edu.cn; fmsong@zju.edu.cn

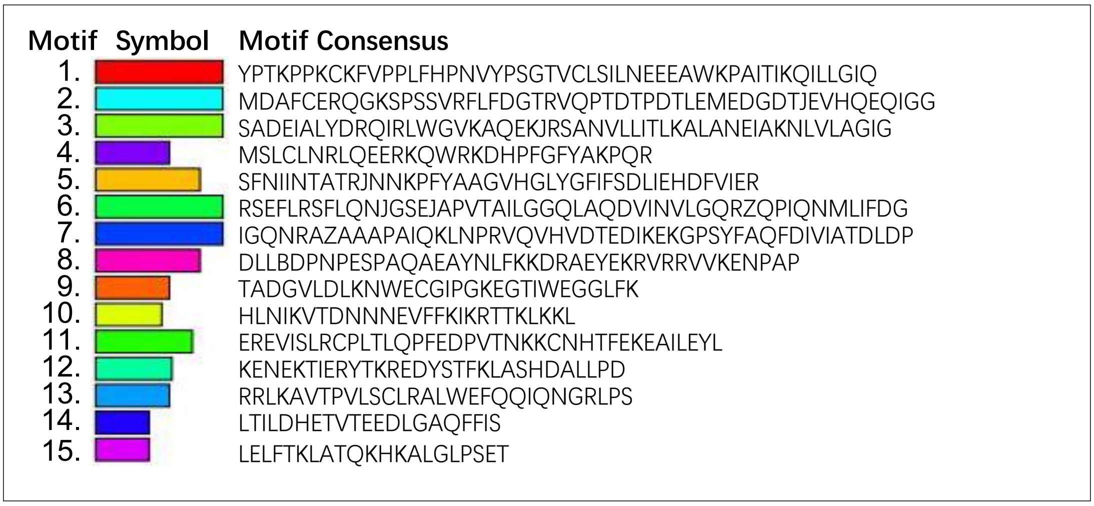

Figure S1. Symbols and consensus sequences of the conserved motifs identified in FonSMT3, FonAOS1, FonUBC9, and FonMMS21 proteins.

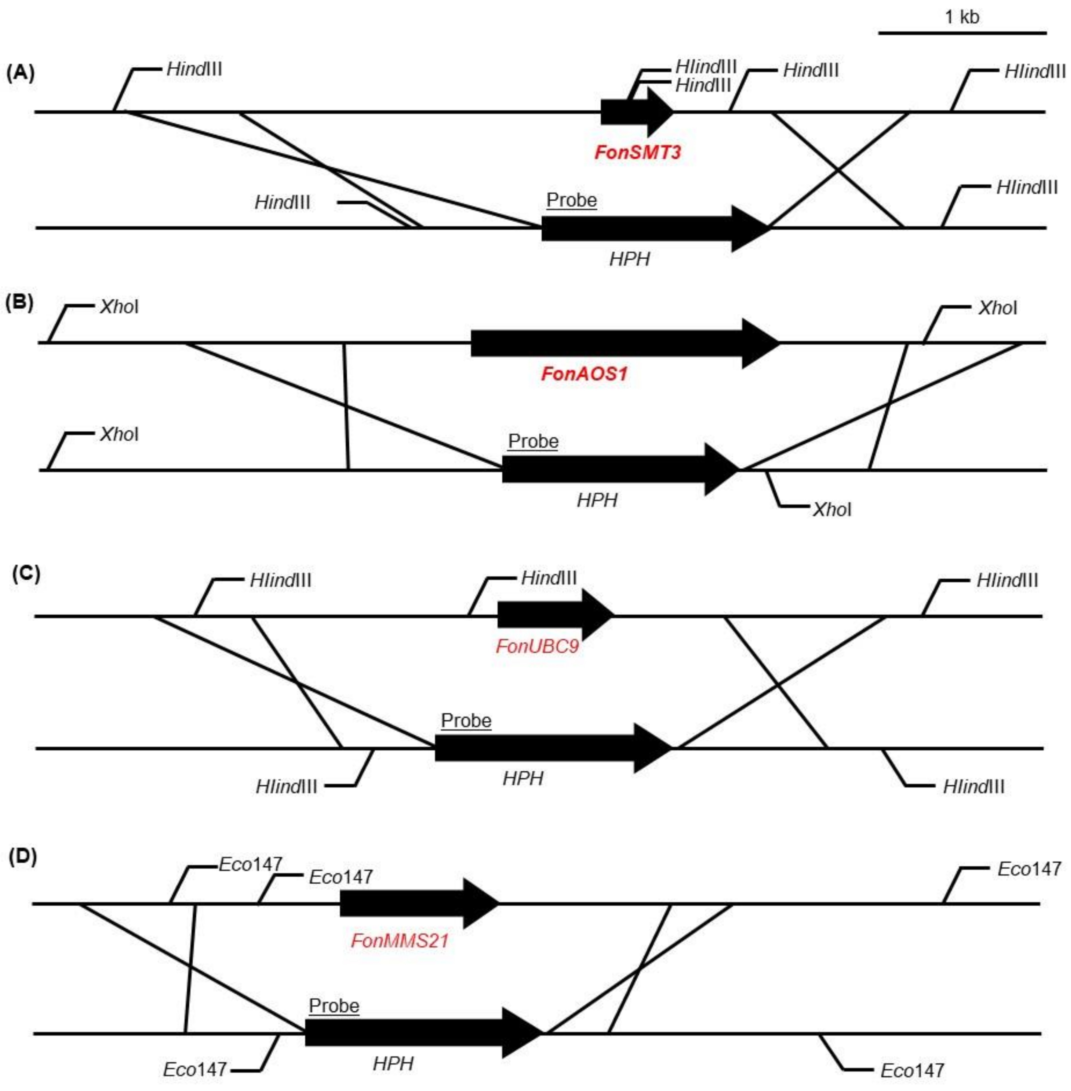

Figure S2. Schematic diagrams of the strategies used to generate the targeted deletion mutants for *FonSMT3* (A), *FonAOS1* (B), *FonUBC9* (C), and *FonMMS21* (D). *HPH*, hygromycin B resistance gene cassette. The *HPH* fragment used as a hybridization probe is indicated.

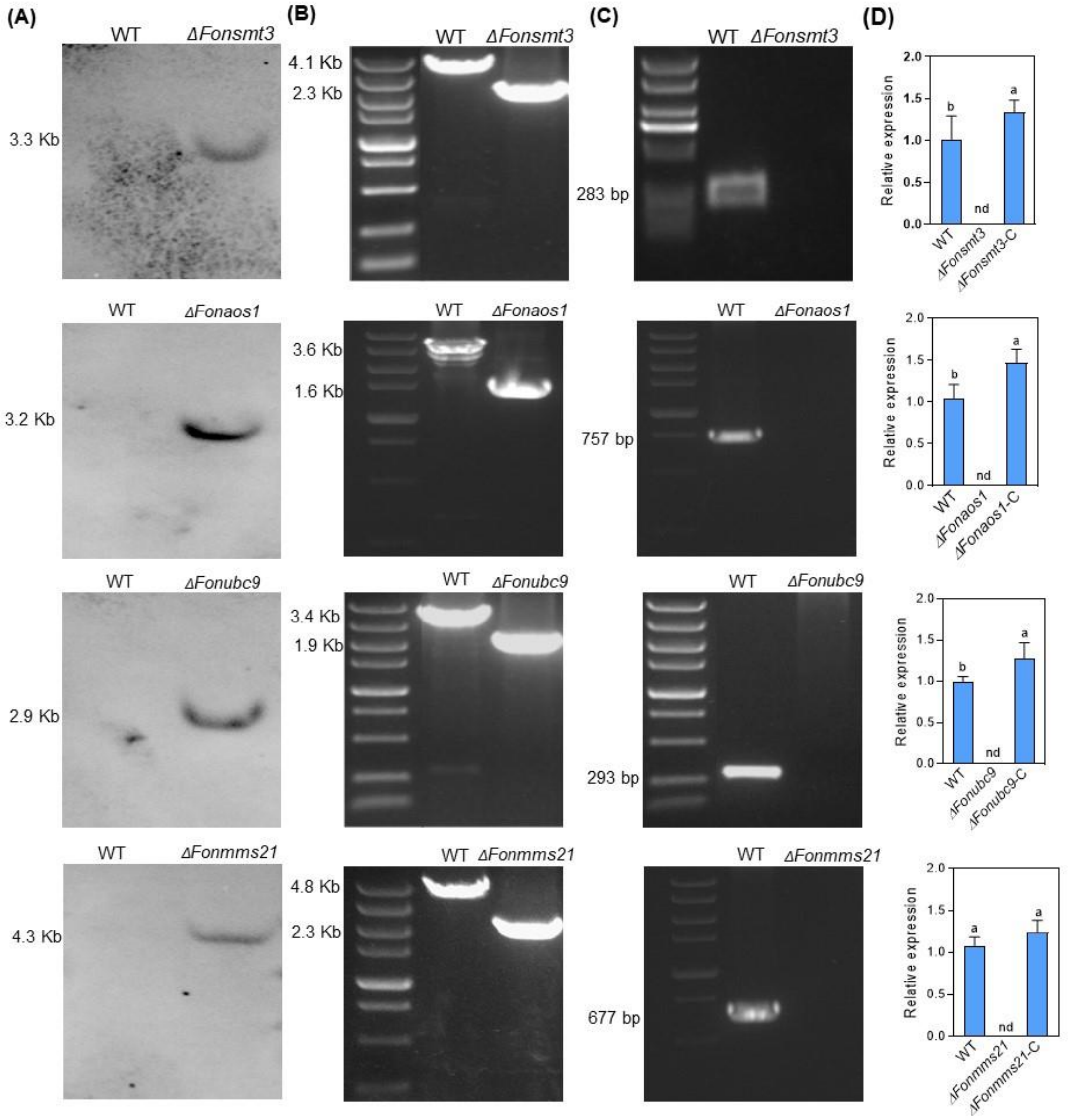

Figure S3. Characterization of the *ΔFonsmt3*, *ΔFonaos1*, *ΔFonubc9*, and *ΔFonmms21* strains. (A) Southern blotting validation of the deletion mutants. Genomic DNA was digested with appropriate restriction enzymes, separated by electrophoresis, and then transferred to Nitrocellulose membranes, followed by hybridization with the DIG-labeled *HPH* fragment probe. (B and C) PCR confirmation of the deletion mutants by amplifying (B) the inserted *HPH* cassette in comparison to WT and (C) gene fragments in WT in comparison to the deletion mutants. (D) RT-qPCR analysis of the transcript levels of the target genes in WT, deletion mutants, and complementation strains. The relative transcript levels in WT was set to 1 and the RT-qPCR data were normalized with *FonActin*. Data presented are the means ±SD from three independent experiments and asterisks above the columns indicate the significant difference at $p<0.05$ level. nd, not detectable.

Table S1. Primers used in this study.

Gene ID	Primer	Sequence (5'-3')
Generation of deletion mutants		
HPH	HPH-F	GGAGGTCAACACATCAATGCCTATT
	HPH-R	CTACTCTATTCCTTTGCCCT
FOMG_00702	SMT3-up-F	TTGATTGAGGAGCGAGTG
	SMT3-up-R	CAAAATAGGCATTGATGTGTTGACCTCCTATTGGGCATGTTTACGG
FOXG_09330	AOS1-up-F	AACTTGGCGGTAGCGGTGAG
	AOS1-up-R	CAAAATAGGCATTGATGTGTTGACCTCCTCGCAGGCACAAAGGGTC
FOXG_05489	UBC9-up-F	CGATTCTTGATAAGCCATTC
	UBC9-up-R	CAAAATAGGCATTGATGTGTTGACCTCCAGTAAACCAGAAGCACCC
FOXG_03253	MMS21-up-F	TGAGGGATGAATGAACTGG
	MMS21-up-R	CAAAATAGGCATTGATGTGTTGACCTCCATCGTGATGGCGGGTAGA
FOXG_07736	UBA2-up-F	CGTTGTTCTGGCTTACCT
	UBA2-up-R	CAAAATAGGCATTGATGTGTTGACCTCCTTTCGCTTGGCTCATTCT
FOXG_20319	SIZ1-up-F	AGAATAGAACAAAGGAGGGA
	SIZ1-up-R	CAAAATAGGCATTGATGTGTTGACCTCCACGATGCCAAGGAGGATG
FOMG_00702	SMT3-down-F	CGTCCGAGGGCAAAGGAATAGAGTAGGAAGGGAAACAGACCAACC
	SMT3-down-R	ACGACAAACCGAATCCAC
FOXG_09330	AOS1-down-F	CGTCCGAGGGCAAAGGAATAGAGTAGTGTTGGGTATCGTAGCAGC
	AOS1-down-R	CGAAGTGGCAAAGAAAGG
FOXG_05489	UBC9-down-F	CGTCCGAGGGCAAAGGAATAGAGTAGCCGCATGTCCGCTGTATT
	UBC9-down-R	AGGGTTGTGCCTTAGTCG
FOXG_03253	MMS21-down-F	CGTCCGAGGGCAAAGGAATAGAGTAGTCTCATTGATAGATTCCTCGTC
	MMS21-down-R	CTTCCAGAATGGCTCGTT
FOXG_07736	UBA2-down-F	CGTCCGAGGGCAAAGGAATAGAGTAGGGCATATTTAGAGGCGAGGAT
	UBA2-down-R	AACCGATTGCTTGGTCAGG
FOXG_20319	SIZ1-down-F	CGTCCGAGGGCAAAGGAATAGAGTAGACCGACACCTGAGTTGAA
	SIZ1-down-R	GCAATGAAGAAGCACGACA
FOMG_00702	SMT3-nest-F	TTGATTGAGGAGCGAGTG
	SMT3-nest-R	ACGACAAACCGAATCCAC
FOXG_09330	AOS1-nest-F	AACTTGGCGGTAGCGGTGAG
	AOS1-nest-R	CGAAGTGGCAAAGAAAGG
FOXG_05489	UBC9-nest-F	TGAGGGATGAATGAACTGG
	UBC9-nest-R	CTTCCAGAATGGCTCGTT
FOXG_03253	MMS21-nest-F	TGAGGGATGAATGAACTGG
	MMS21-nest-R	CTTCCAGAATGGCTCGTT
FOXG_07736	UBA2-nest-F	CGTTGTTCTGGCTTACCT
	UBA2-nest-R	AACCGATTGCTTGGTCAGG
FOXG_20319	SIZ1-nest-F	AGAATAGAACAAAGGAGGGA
	SIZ1-nest-R	GCAATGAAGAAGCACGACA

Identification of deletion mutants		
FOMG_00702	SMT3-ID1-F	TCTCGTCGTTCTTCGTTT
	SMT3-ID1-R	ATTCCATCCGCAGTGTTT
FOXG_09330	AOS1-ID1-F	ACCCAGTTAGCCTCGTTC
	AOS1-ID1-R	CTCACCCGCTGTTTCTTT
FOXG_05489	UBC9-ID1-F	CTCCATAGTTCGCTTGATAG
	UBC9-ID1-R	TCCACCTTCCTCGCTTGT
FOXG_03253	MMS21-ID1-F	TTATTGGGAGTCAAGTAGAGC
	MMS21-ID1-R	CCGAAAGTTATGGTGGTC
FOXG_07736	UBA2-ID1-F	GTATTAGCTTGCTATGCGTGAG
	UBA2-ID1-R	GCGGCTCTGTTGTAACCATC
FOXG_20319	SIZ1-ID1-F	CTGCTAAACGCCGATGAA
	SIZ1-ID1-R	TGGCTCGCCGTATTGTCT
FOMG_00702	SMT3-ID2-F	CAACAACGAGGTCTTCTTCA
	SMT3-ID2-R	TATCGCCATCCTGCATCT
FOXG_09330	AOS1-ID2-F	CTGTCAGTGCTGGTAAGTCC
	AOS1-ID2-R	TGAGTCGTCGCTTGGATT
FOXG_05489	UBC9-ID2-F	TTGGTCTGGTGGACTCTT
	UBC9-ID2-R	GGATTTGTTTGACGGTGAT
FOXG_03253	MMS21-ID2-F	ATTACTTGGCTTTAGCGTCAG
	MMS21-ID2-R	TCCCTCTTGAGCGGTCTT
FOXG_07736	UBA2-ID2-F	AGCAAGAGCAGCAGCAAA
	UBA2-ID2-R	CCGTTGTCGGCATCAGTT
FOXG_20319	SIZ1-ID2-F	CGCCCGAAATACACCAAC
	SIZ1-ID2-R	TTGCCTGAGTATCCGTTC
HPH probe	HPH-Probe-F	GGTTCATTTAGGCAACTGGT
	HPH-Probe-R	TGTAGTGTATTGACCGATTCC
Construction of the complementation strains		
FOMG_00702	SMT3-nPYF111p1-F	TTTCGTAGGAACCCAATCTTCAAAATGGTGAGCAAGGGCGAGGAG
	SMT3-nPYF11p1-R	CTCGCCAGGAGTTCCGTTTTCGTTCTCGTTGGACATCTTGTACAGCTCGTCCAT
	SMT3-nPYF11p2-F	ATGTCCAACGAGAACGAAAA
	SMT3-nPYF11p2-R	GTGCGCAGAGGAGCCTGAATGTTGAGTGGAATGATGTTACTGTGCAGATCCGCCC
	SMT3-nPYF11-nest-F	TTTCGTAGGAACCCAATCTTCAAAATGGTGAGCAAGGGCGAGGAG
	SMT3-nPYF11-nest-R	GTGCGCAGAGGAGCCTGAATGTTGAGTGGAATGATGTTACTGTGCAGATCCGCCC
FOXG_09330	AOS1-PYF11-F	TTTCGTAGGAACCCAATCTTCAAAATGGACAACTCGAACCAGGA
	AOS1-PYF11-R	CACCACCCCGGTGAACAGCTCCTCGCCCTTGCTCACCTCTTTCGATTCCGAGGCA
FOXG_05489	UBC9-PYF11-F	TTTCGTAGGAACCCAATCTTCAAAATGGCGCTTTGCCAGAACCGA
	UBC9-PYF11-R	CACCACCCCGGTGAACAGCTCCTCGCCCTTGCTCACAGGTGTGGGGTTCTCGCGAA
FOXG_03253	MMS21-PYF11-F	TTTCGTAGGAACCCAATCTTCAAAATGTCTCGGAGAGGTATCAA

	MMS21-PYF11-R	CACCACCCCGGTGAACAGCTCCTCGCCCTTGCTCACTTCTCCTCTCTTCTCCCTCT
RT-qPCR		
FOMG_00702	SMT3-RT-F	GCTTATGGGCGCTTTTTGCG
	SMT3-RT-R	TATCGGTGGGTTGGACTCGC
FOXG_09330	AOS1-RT-F	CCTGGGATGGGCTTACCGAAT
	AOS1-RT-R	TACAAAGCGACCTCGTCAGCA
FOXG_05489	UBC9-RT-F	CTAACCCCGAGTCACCTGCC
	UBC9-RT-R	ACTCGCTTCTCGTACTCGGC
FOXG_03253	MMS21-RT-F	CAGAGCCGAATTGGAAGACGAAG
	MMS21-RT-R	TCGCCTTGTTGGGTCTCCA
FOXG_01569	FonActin-RT-F	GAGGGACCGCTCTCGTCGT
	FonActin-RT-R	GGAGATCCAGACTGCCGCTCAG

Table S2. The SUMOylation pathway components in *Fusarium oxysporum* and their similarity to the orthologs in *M. oryzae*.

Components	Proteins	*F. oxysporum*	*M. oryzae*	Similarity	PF Domain
SUMO	SMT3	FOXG_00063	MGG_05737	76%	SUMO (PF11976)
E1	AOS1	FOXG_09330	MGG_01669	55%	Thif (PF00899)
	UBA2	FOXG_07736	MGG_06733	61%	Thif (PF00899), UAE (PF14732), UBA (PF10585)
E2	UBC9	FOXG_05489	MGG_00970	85%	ubiquitin-conjugating domain (PF00179)
E3	SIZ1	FOXG_20319	MGG_08837	51%	zinc finger domains (PF14324), (PF02891)
	MMS21	FOXG_03253	MGG_0104)	35%	SP-RING-type (PF11789)

Table S3. Gene structure and motif organization of SMT3, AOS1, UBC9, and MMS21 in *Fusarium oxysporum*, *Homo sapiens*, *Phytophthora infestans*, and other fungi.

Organisms	Accessions	Genomic sequence (bp)	CDS (bp)	Exon/ intron	MEME motifs		Best hits by SMART
					Uncharacterized	Characterized	
**SMT3**							
Fusarium oxysporum	XP_018231639.1	1600	297	2/1	10	2 (ubiquitin)	UBQ
Fusarium proliferatum	XP_031075851.1	410	297	2/1	10	2 (ubiquitin)	UBQ
Magnaporthe oryzae	XP_003710648.1	443	330	2/1	10	2 (ubiquitin)	UBQ
Colletotrichum graminicola	XP_008093238.1	367	303	3/2	10	2 (ubiquitin)	UBQ
Botrytis cinerea	XP_024551758.1)	1025	291	2/1	10	2 (ubiquitin)	UBQ
Aspergillus flavus	XP_041149378.1	347	279	2/1	10	2 (ubiquitin)	UBQ
Aspergillus nidulans	XP_658795.1	383	285	2/1	10	2 (ubiquitin)	UBQ
Blumeria graminis	EPQ65659.1	397	312	2/1	10	2 (ubiquitin)	UBQ
Saccharomyces cerevisiae	NP_010798.1	306	306	1/0	10	2 (ubiquitin)	UBQ
Schizosaccharomycs pombe	AAB71541.1	497	354	5/4	~	2 (ubiquitin)	UBQ
Candida albicans	XP_713803.1	309	309	1/0	10	2 (ubiquitin)	UBQ
Phytophthora infestans	XP_002903234.1	306	306	1/0	~	2 (ubiquitin)	UBQ
Homo sapiens	CAA67896.1	12446	312	4/3	~	2 (ubiquitin)	UBQ
**AOS1**							
Fusarium oxysporum	XP_018246510.1	2536	1719	2/1	14,5,12,13,15	3 (ThiF), 7 (ThiF), 6 (3KYD|A)	Thif
Fusarium proliferatum	XP_031081694.1	1771	1716	2/1	14,5,12,13,15	3 (ThiF), 7 (ThiF), 6 (3KYD|A)	Thif
Magnaporthe oryzae	XP_003714655.1	2749	1350	2/1	14,5,12,13,15	3 (ThiF), 7 (ThiF), 6 (3KYD|A)	Thif
Colletotrichum graminicola	XP_008092598.1	1588	1533	2/1	14,5,12,13,15	3 (ThiF), 7 (ThiF), 6 (3KYD|A)	Thif
Botrytis cinerea	EMR89522.1	1954	1206	2/1	14,5,12,13,15	3 (ThiF), 7 (ThiF), 6 (3KYD|A)	Thif

Aspergillus flavus	RAQ50318.1	1387	1185	4/3	14,5,12,13,15	3 (ThiF), 7 (ThiF), 6 (3KYD|A)	Thif
Aspergillus nidulans	XP_659902.1	1314	1191	3/2	14,5,12,13,15	3 (ThiF), 7 (ThiF), 6 (3KYD|A)	Thif
Blumeria graminis	EPQ63982.1	1284	1230	2/1	5,12,13, 14,15	3 (ThiF), 7 (ThiF), 6 (3KYD|A)	Thif
Saccharomyces cerevisiae	EGA76517.1	1040	1044	1/0	5,14,	3 (ThiF), 7 (ThiF)	Thif
Schizosaccharomycs pombe	NP_593251.1	1510	924	5/4	14,5	3 (ThiF), 7 (ThiF), 6 (3KYD|A)	Thif
Candida albicans	KGQ83238.1	1170	1170	1/0	14,5	3 (ThiF), 6 (3KYD|A)	Thif
Phytophthora infestans	XP_002896819.1	1132	954	4/3	14,5	3 (ThiF), 7 (ThiF), 6 (3KYD|A)	Thif
Homo sapiens	NP_001139185.1	79801	900	7/6	14,5	3 (ThiF), 7 (ThiF)	Thif
**UBC9**							
Fusarium oxysporum	XP_018240801.1	2646	474	4/3	4,9	1(UQ_con), 8(UQ_con)	UBCc
Fusarium proliferatum	KAG4264306.1	631	474	4/3	4,9	1(UQ_con), 8(UQ_con)	UBCc
Magnaporthe oryzae	XP_003717980.1	1656	474	4/3	4,9	1(UQ_con), 8(UQ_con)	UBCc
Colletotrichum graminicola	XP_008092998.1	664	477	4/3	4,9	1(UQ_con), 8(UQ_con)	UBCc
Botrytis cinerea	XP_001557575.1	1105	477	4/3	4,9	1(UQ_con), 8(UQ_con)	UBCc
Aspergillus flavus	KAB8246909.1	728	474	4/3	4,9	1(UQ_con), 8(UQ_con)	UBCc
Aspergillus nidulans	AAX89142.1	1652	474	4/3	4,9	1(UQ_con), 8(UQ_con)	UBCc
Blumeria graminis	EPQ63458.1	569	450	2/1	4,9	1(UQ_con), 8(UQ_con)	UBCc
Saccharomyces cerevisiae	NP_010219.1	584	474	2/1	4,9	1(UQ_con)	UBCc
Schizosaccharomycs pombe	NP_593204.1	1286	474	6/5	4,9	1(UQ_con)	UBCc
Phytophthora infestans	XP_002903310.1	483	483	1/0	4,9	1(UQ_con), 8(UQ_con)	UBCc
Homo sapiens	2GRR_A	17886	516	7/6	4,9	1(UQ_con), 8(UQ_con)	UBCc
**MMS21**							
Fusarium oxysporum	XP_018237250.1	1586	1125	2/1	12	zf-RING_UBOX	zf-Nse
Fusarium proliferatum	XP_031082828.1	1175	490	2/1	12	zf-RING_UBOX	zf-Nse
Magnaporthe oryzae	XP_003717900.1	1972	1245	2/1	12	zf-RING_UBOX	zf-Nse
Colletotrichum graminicola	XP_008094278.1	1306	1254	2/1	12	zf-RING_UBOX	zf-Nse

Botrytis cinerea	XP_001555237.1	1603	1290	2/1	12	zf-RING_UBOX	zf-Nse
Aspergillus flavus	KOC10680.1	1553	1461	2/1	12	zf-RING_UBOX	zf-Nse
Aspergillus nidulans	CBF85821.1	1815	1509	2/1	12	zf-RING_UBOX	zf-Nse
Blumeria graminis	EPQ67616.1	1256	1086	4/3	12	zf-RING_UBOX	zf-Nse
Saccharomyces cerevisiae	7P47_A	804	804	1/0	0	zf-RING_UBOX	zf-Nse
Schizosaccharomycs pombe	NP_001343015.1	1405	753	3/2	12	zf-RING_UBOX	zf-Nse
Phytophthora infestans	XP_002906313.1	996	729	5/4	0	zf-RING_UBOX	zf-RING_UBOX
Homo sapiens	NP_001336414.1	8306	744	6/5	0	zf-RING_UBOX	zf-RING_UBOX
